# Immunomodulatory effect of statins on Regulatory T Lymphocytes in human colorectal cancer is determined by the stage of disease

**DOI:** 10.18632/oncotarget.26293

**Published:** 2018-11-06

**Authors:** Belal A. Al-Husein, Bara’ Dawah, Saleem Bani-Hani, Samir M. Al Bashir, Khaled M. Al-Sawalmeh, Nehad M. Ayoub

**Affiliations:** ^1^ Department of Clinical Pharmacy, Faculty of Pharmacy, Jordan University of Science and Technology, Irbid, Jordan; ^2^ Department of Medical Laboratory Sciences, Faculty of Applied Medical Sciences, Jordan University of Science and Technology, Irbid, Jordan; ^3^ Department of Pathology, Faculty of Medicine, Jordan University of Science and Technology, Irbid, Jordan

**Keywords:** colorectal cancer, disease stage, statins, Regulatory T Lymphocytes, TGF-beta

## Abstract

Colorectal cancer (CRC) is a public health problem worldwide and in Jordan. Statins are cholesterol lowering agents. Beyond their effects, statins use has been reported to reduced risk of several malignances, including CRC. This study aimed to assess the effect of statins on CRC by studying cellular infiltration of Regulatory T Lymphocytes (Tregs) into CRC tissues and their effect on Transforming growth factor beta 1 (TGF-β_1_) level and on angiogenesis. Fourty seven specimens (25 statins users vs. 22 non-users) were used. Immunohistochemistry was performed to study Tregs infiltration using their marker, fork head transcription factor, and angiogenesis using CD31 as a marker. TGF-β_1_ levels were measured using ELISA. Results revealed that statins use was associated with more Tregs infiltration, less angiogenesis but no difference in TGF-β_1_ content in tumor tissue. When results were further stratified according to stage of disease, more Tregs infiltration was significantly noticed in advanced disease but not in early disease. In addition, more angiogenesis inhibition was noticed in early disease but not in advanced disease. Same stage-dependence wasn’t noticed with TGF-β_1_ expression. In early disease, reduction of angiogenesis mediated by statins might lead to reduction of tumor aggressiveness. On the other hand, Tregs infiltration into tumor mediated by statins might reduce cancer aggressiveness in advanced disease. These results suggest that statins might be used in the treatment of CRC.

## INTRODUCTION

Colorectal cancer (CRC) is the third most common cancer and the second leading cause of mortality among cancers in the United States [1]. In Jordan, it is the second most common cancer after breast cancer [2]. Many overlapping etiologies have been linked to the incidence of this cancer, such as hereditary, genetic and more recently, immunologic [3, 4]. The theory about cancer immunosurveillance, which sets that malignant cells are recognized as foreign and then eliminated by immune system, has been changed after well understanding of immunity and improving animal models and different techniques [5]. In animal models, the interaction between tumors and immune system starts an “immunoediting” process [6]. This process leads to three outcomes: cancer elimination, equilibrium, and escape [7]. Immune evasion plays an important role in immunologic mechanisms of development and progression of cancer [8]. Regulatory T Lymphocytes (also known as Tregs) are one of many major players in tumor evasive mechanisms [9]. Immunity can be suppressed by tumor either systematically or in the tumor microenvironment [5]. Immunosuppressive effects in the microenvironment appears by Tregs domination at which they produce Transforming growth factor beta (TGF-β) and interlukin-10, which are immunosuppressive cytokines, in particular to effector T cells [10]. Tumors produce TGF-β to escape immune cells destruction via skewing immune response and shifting antitumor effector T cells into Tregs [11]. Systemic immunosuppressive effects of a tumor can be achived through increasing the number of activated granulocytes and myeloid-derived suppressor cells [5]. Surprisingly, an overwhelming evidence links Tregs infiltration into CRC tissue with favorable prognosis [12].

Statins are HMG-CoA reductase inhibitors that inhibit the key step of cholesterol synthesis and are used to treat hypercholesterolemia. In addition to that, statins have some pleiotropic effects [13]. Many studies have linked statins use with a reduction of both incidence and progression of many cancers, including breast [14, 15], prostate [16, 17] and CRC [18, 19] as well. Some evidence, however, shows neutral effect of statins on those cancers. Statins are being investigated as anticancer agents in many cancers, including cancers of breast, gastric, prostate, lung, colon and in hematologic malignancies [20]. Statins mediate anticancer effects through their antiproliferative, anti-invasive [21, 22], antiangiogenic [23] and proapoptotic effects [21]. Statins inhibit tumor cells proliferation by their effect on cell cycle by blocking the transition from G1 to S phase, this arrest is mediated by downregulation of cyclin-dependent kinase-2 (CDK-2) activity, and upregulation and stabilization of CDK inhibitors p21 and p27 [21, 22]. Apoptosis induction by statins appears to be mediated by downregulation of bcl-2 expression, activation of caspases, cleavage of PARP, and depletion of geranylgeranylated proteins (downstream product of mevalonate pathway) [23]. Also, statins induce apoptosis by increasing the expression of Bax, which is a pro-apoptotic protein [21]. Statins act as anti-invasive agents through inhibition of signaling pathways that are related to cancer invasive properties [21]. Statins angiogenic effects are biphasic: at high statins concentrations angiogenesis will be inhibited, while at low concentrations, they act as pro-angiogenic [24]. Statins inhibit angiogenesis through their ability to downregulate vascular endothelial growth factor (VEGF), which is the major angiogenic mediator and also a tumor growth promotor [23]. Angiogenesis is a main mediator of cancer cells proliferation and dissemination or metastasis. TGF-β is a major angiogenic factor and is a signature secretory component of Tregs.

We have previosuly shown a direct anti-cancer effect for statins on prostate cancer cells, both *in-vitro* and *in-vivo* [25, 26]. Because of their immunomodulatory effect, we predict that statins influence Tregs function and localization. In this research, we explored the effect of statins therapy on Tregs infiltration into CRC tissue, as well as its effect on TGF-β content and vessel content within tumors.

## RESULTS

A total 47 samples from CRC patients aged between 50 and 91 years were included in this study. A total of 25 samples were for statins users (1 sample for 40 mg atorvastatin user, 22 samples for 20 mg atorvastatin users, and 2 samples for 10 mg atorvastatin users), and 22 for statins non-users. Table 1 demonstrates the demographic characteristics of the study population.

**Table 1:** Demographics and clinical characteristics of the study population

Characteristics		Statins users	Statins non-users
Gender	Male Female	16 9	17 5
Age (Average ± SEM)		66.81 ± 1.86	58.35 ± 2.75
Tumor stage	Stage I Stage II Stage III Stage IV	1 4 15 5	1 4 14 3

**Figure 1:**
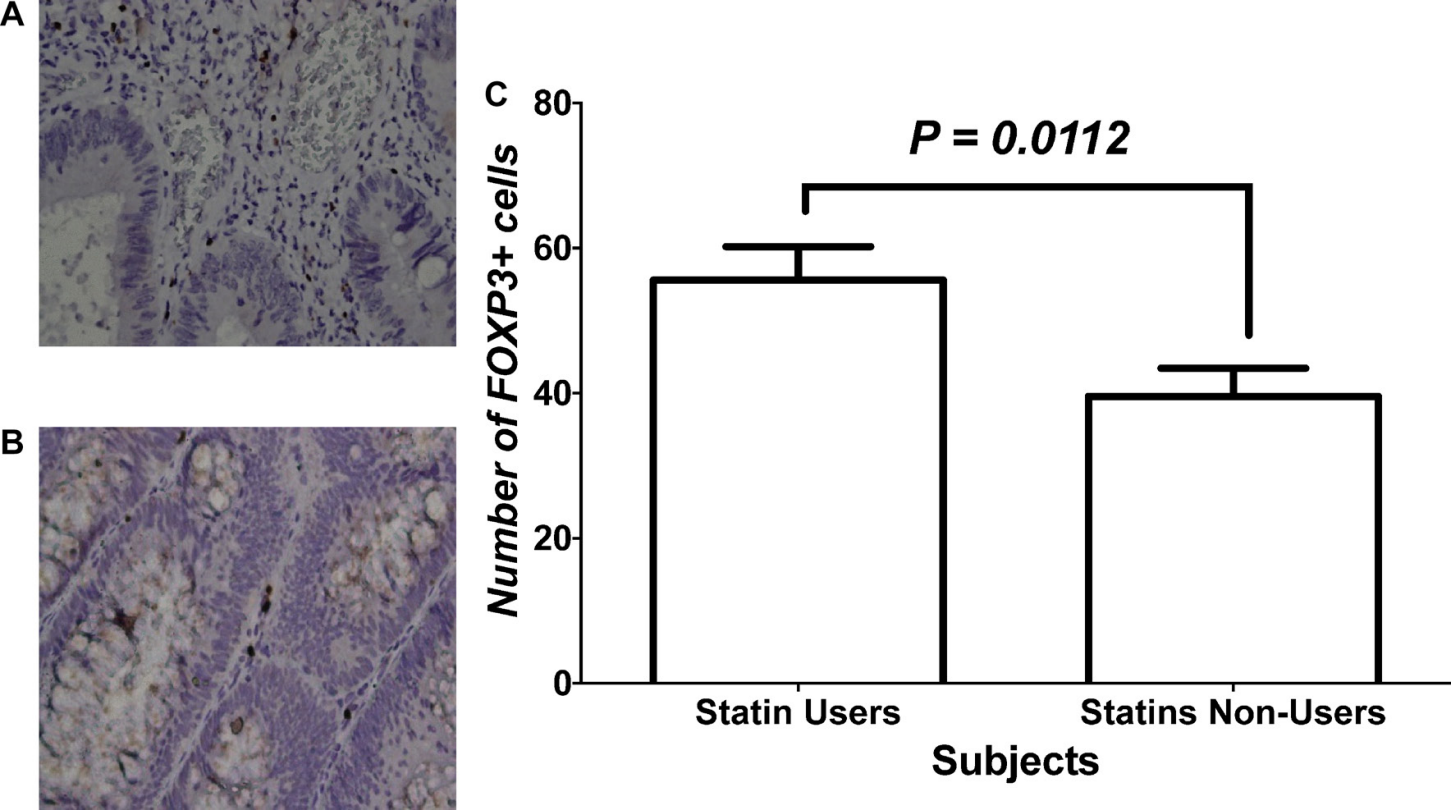
Immunohistochemical analysis of FOXP3+ cells within CRC tissues of study groups (**A** and **B**) represent immunohistochemical staining with FoxP3 for tissues of patients who were statins users vs. statins non-users, respectively. (**C**) Statistical analysis of the two treatment groups.

Figure 1A and 1B, show immunohistochemical staining of FoxP3+ Tregs infiltration into human CRC tissues in statins users and non-users, respectively. Figure 1C shows quantification of FoxP3+ Tregs within tissues in both study groups. It demonstrates the number of FoxP3 positive cells that infiltrated tumor tissues of statins users compared to statins non-users. There was a significantly greater density of infiltrating Tregs among statin users compared to non-users as indicated by positive staining of FoxP3.

**Figure 2:**
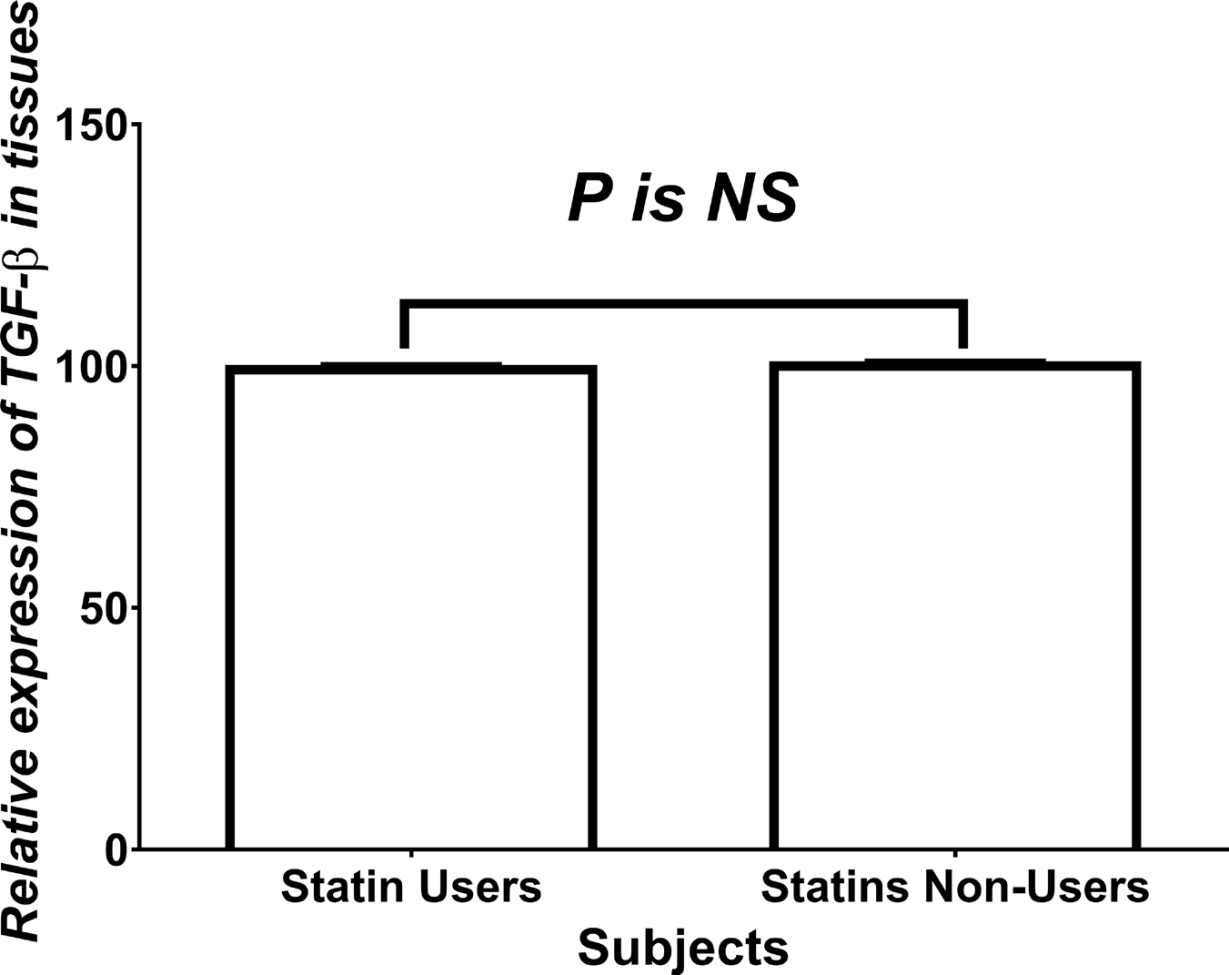
Relative expression of TGF-β NS = statistically non-significant.

Figure 2 shows the relative expression of TGF-β_1_ in tissues of both statins users and non-users among CRC patients. No significant difference between users and non-users of statins was found regarding total levels of TGF-β in CRC tissues.

**Figure 3:**
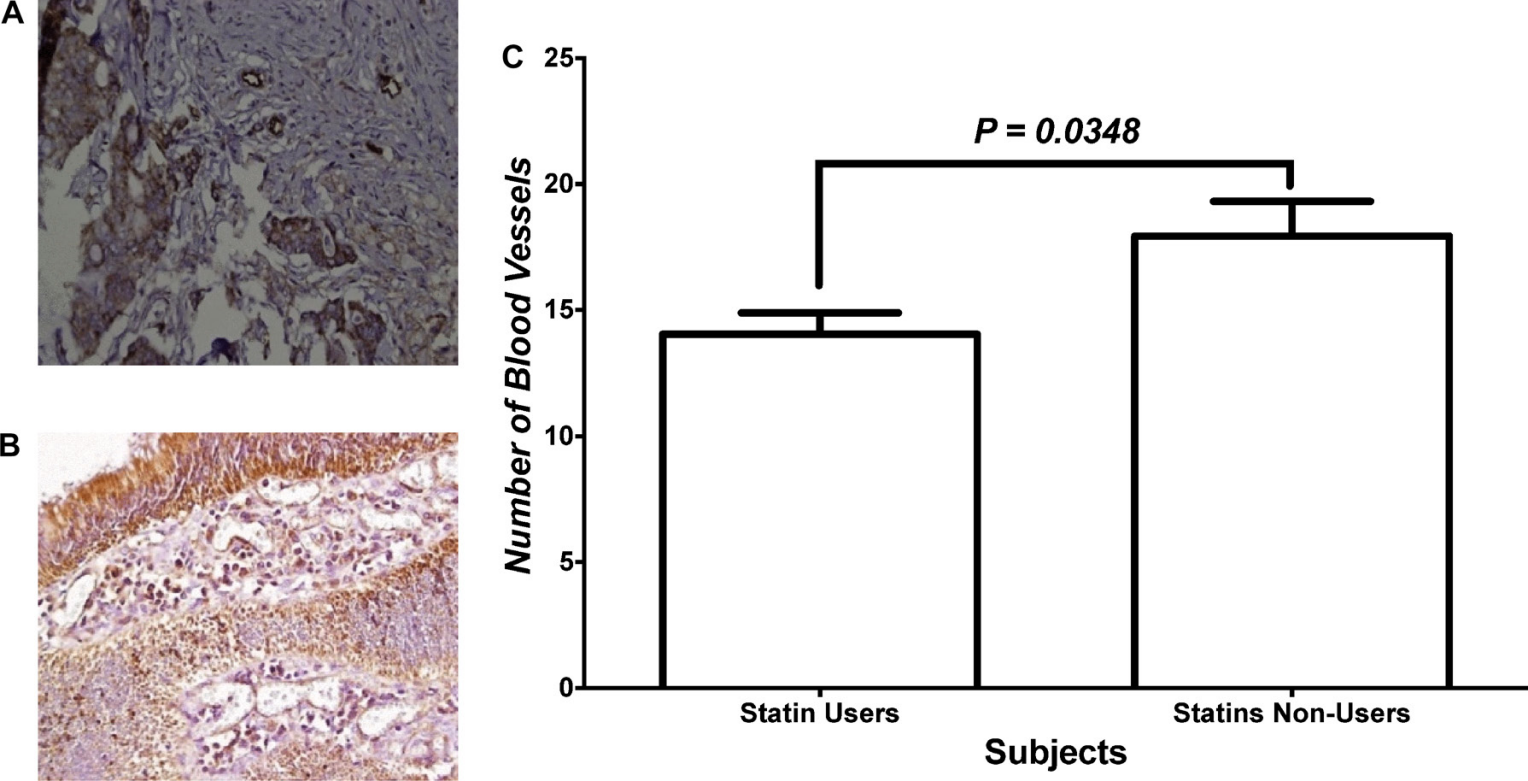
Angiogenesis within tumor tissues (**A** and **B**) Immunohistochemical staining with CD31 for tissues of patients who were statins users vs. statins non-users, respectively. (**C**) Statistical analysis of the two treatment groups.

Figure 3A and 3B show the immunohistochemical analysis of vascularity within tumor tissues of statin users and non-users, respectively. Tumor sections were stained for CD31 which is a marker of vascularity. Figure 3C shows the number of blood vessels within tumor tissues in both study groups. The number of blood vessels, as indicated by positive CD31 staining, was significantly lower among statins users compared to non-users.

**Figure 4:**
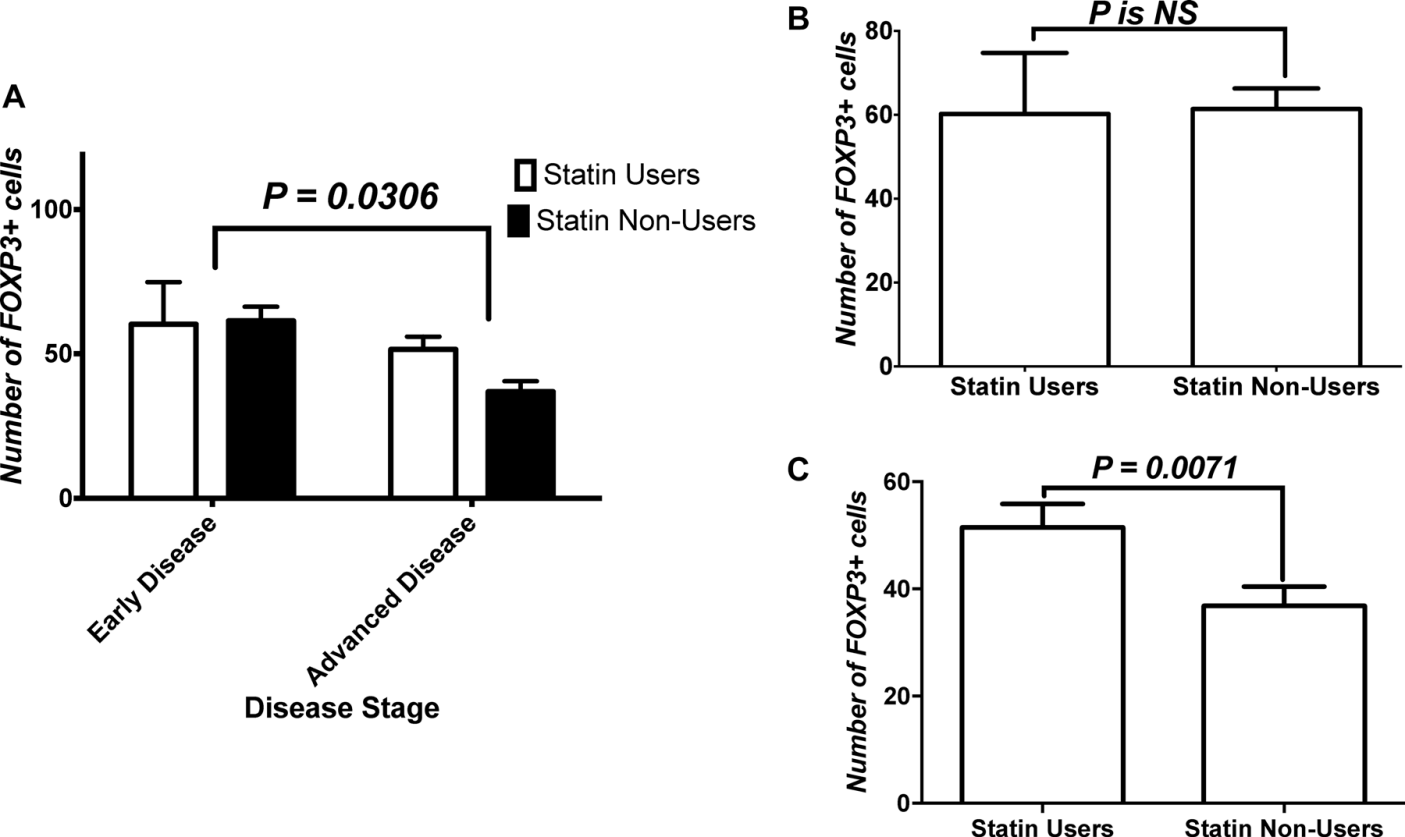
Effect of stage on FoxP3+ cells tumor infiltration (**A**) Analysis for the impact of disease stage and treatment on number of FOXP3+ cells infiltrating tumors. (**B**) Analysis of FoxP3+ cells infiltration within tissues of patients with early disease, and (**C**) that within tissues of patients with advanced disease. NS = statistically non-significant.

Figure 4A shows the analysis for the impact of disease stage and treatment on number of FOXP3+ cells infiltrating tumors. Two-Way ANOVA showed an effect of disease stage on the infiltration of FoxP3+ Tregs within tumors, but not treatment or interaction. Within these groups, we performed a subanalysis of changes within the early and advanced disease groups. Mann-Whitney *U* test failed to show a difference between both treatment groups. Figure 4B shows the analysis of FoxP3+ cells infiltration within tumor tissues of patients with early disease while Figure 4C shows that within tissues of patients with advanced disease. Mann-Whitney *U* test showed that CRC tissue of statins users in advanced disease had more FoxP3+ Tregs infiltration compared to non-users’ CRC tissues.

**Figure 5:**
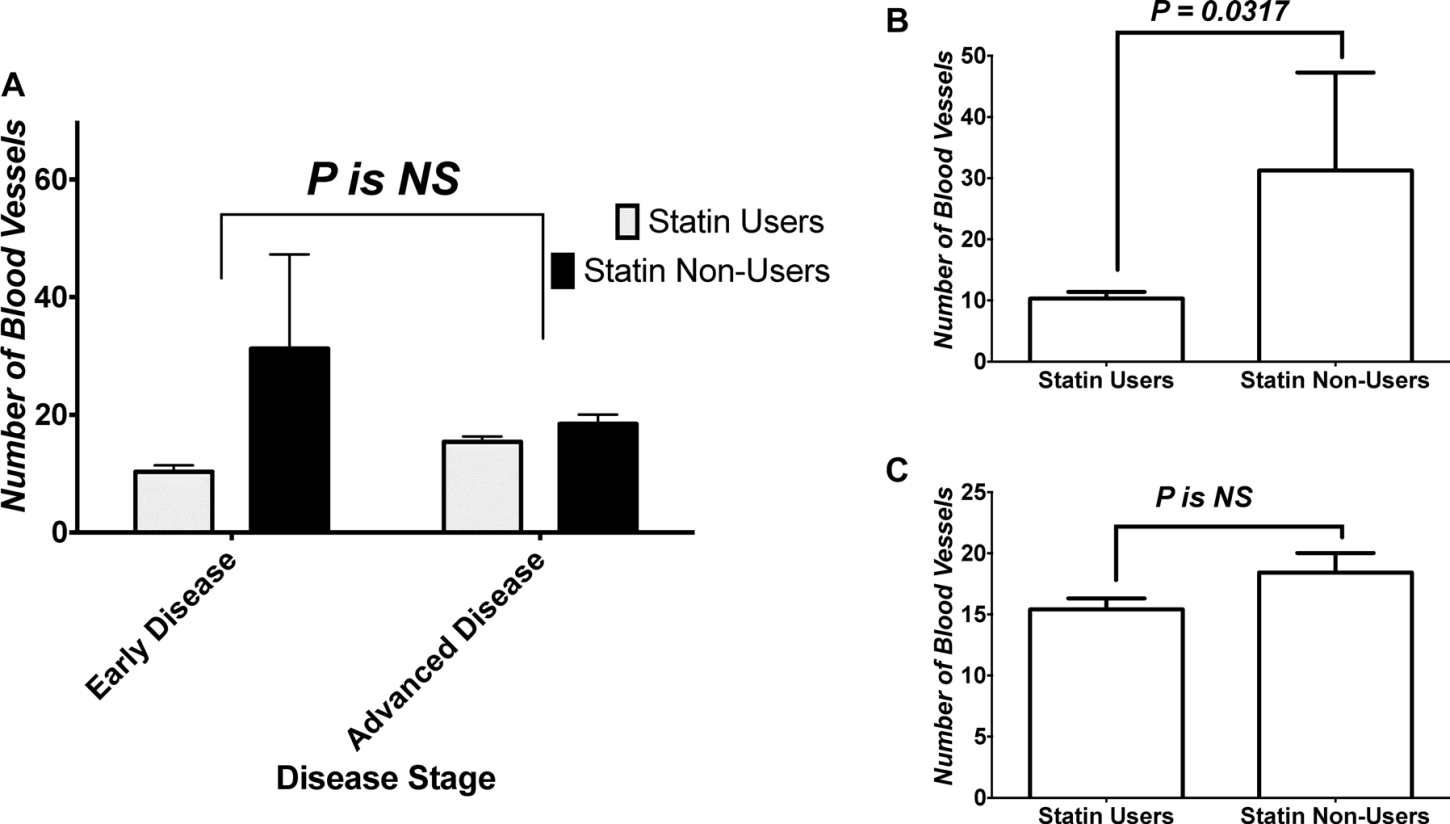
Effect of stage on angiogenesis within tumor (**A**) Analysis of impact of disease stage and treatment on number of blood vessels within tumors. (**B**) Analysis of vessel content within different treatment groups’ tumor tissues in early disease, and (**C**) vessel content comparison between both treatment groups in advanced disease. NS = statistically non-significant.

Figure 5A shows the analysis of impact of disease stage and treatment on number of blood vessels within tumors. Two-Way ANOVA showed an effect of both the treatment and stage-treatment interaction on vessel content of tissues, but not an effect of stage. Within those groups, we performed a subanalysis of changes within the early and advanced diease groups. Figure 5B shows the analysis of vessel content within different treatment groups’ tumor tissues in early disease. Mann-Whitney *U* test showed that CRC tissue of statins users in early disease had significantly less number of vessels compared to non-users’ CRC tissues. On the other hand, Figure 5C shows vessel content comparison between both treatment groups in advanced disease. Mann-Whitney *U* test failed to show a difference between both treatment groups in advanced disease.

## DISCUSSION AND CONCLUSIONS

Statins are known to have anticancer effects. For that reason, their use might help to reduce the risk of many cancers including CRC. This research questions the significance of use of statins, a commonly prescribed class of medications, concomitantly in CRC patients.

This study revealed that statins treatment leads to a significant higher density of FoxP3+Tregs infiltration within tumor tissues of CRC patients. Similar effect of statins have been reported in a study that showed an increase in Tregs frequency in healthy individuals as part of statins’ immunomodulatory effects [29]. Another study showed that statins use increases the frequency and suppressor effects of Tregs in hypercholesterolemia subjects. The same study revealed that statins use significantly leads to an increase in the number of FoxP3+ T cells in the peripheral blood [30]. Similar effect of statins has been observed in a study in rheumatoid arthritis patients [31]. In addition to that, simvastatin use significantly increases the number of FoxP3+ Tregs expression in atherosclerotic plaques, also it increases the suppressive function of Tregs, this finding has been reported by another study [32]. Increased content of Tregs within CRC tissue is of important prognostic value as indicated by others [33]. Researchers suggest the effect to be mediated through suppression of T_H_17 cells [34].

In this study, there were no significant differences between statins users and non-users in regard to TGF-β expression. Some studies have shown that statins reduce TGF-β expression in diabetic animals’ tissue [35, 36]. A study was conducted on rats has reported that rosuvastatin, in a dose-dependent manner, reduced the expression of TGF-β_1_ in diabetic cardiomyopathy [35]. Another study on diabetic rats showed that lovastatin significantly inhibits the expression of TGF-β_1_ [36]. However, some studies have reported that statins induce TGF-β expression. Simvastatin significantly induces the expression of TGF-β, according to a study that was performed on rats mesangial cells [37]. Similar finding has been revealed by another study. It has been shown that significant downregulation of TGF-β_1_ by simvastatin can be protective in diabetic rats kidneys [38]. Also, a study was conducted on mice has shown that TGF-β expression was significantly increased by simvastatin in atherosclerotic plaque [32]. Pravastatin has also the same effect in hypercholesterolemic patients, as it significantly increased plasma level of TGF-β_1_ [39]. Another study showed similar findings in blood of patients with ST-segment elevated myocardial infarction reciving 80 mg of oral atorvastatin before primary percutaneous coronary intervention in regard to mRNA expression of both TGF-β and FoxP3 expression [40]. These findings somehow contradict the results of the present study. However, we should take into consideration that tumor microenvironment is different from any of the aforementioned tissues.

Furthermore, a significant reduction in CD31 expression within tumor tissues mediated by statins is another important finding in this study. This translates to a significant decrease of tumor vessel content and thus angiogenesis. Many studies have demonstrated that statins effect on angiogenesis depends on their concentration in the blood or their dose. Low doses of statins induce angiogenesis, while at high doses they become angiostatic [24, 41, 42]. This finding is consistent with the result of this study because moderate doses of atorvastatin were used in the enrolled subjects. Other study has demonstrated that statins induce angiogenesis in different mechanism at which low doses of atorvastatin increased VEGF expression, as evident in a study conducted on a rat model [43]. Another study has shown that treatment with low dose of atorvastatin leads to a significant increase of VEGF-induced angiogenic responsiveness of coronary endothelial cells in normal and diabetic rats [44]. Another study conducted on simvastatin has reported the same results in which simvastatin has enhanced myocardial angiogenesis through increased VEGF expression levels [45].

Little is known about the relationship between colon cancer stage (early vs. advanced) and Tregs infiltration or level of blood vessels. Only one study found that FoxP3+ cell infiltration into lymphoid follicles from histologically normal mucosa as a prognostic factor in colon cancer at an early stage [46]. This study is not even discussing the tumor content of FoxP3+ Tregs. In regard to angiogenesis, one study showed that the expression of VEGF-1 increases significantly at stage IV of disease, compared to stages II and III [47]. This is though not a direct measure of vascularity of tumor tissue. Despite that, we predict that higher FoxP3+ cells within the tumor in advance stages is important in suppression of tumor dissemination and proliferation through suppression of inflammatory processes. This could probably be through T_H_17 suppression [34] or by inflammasome-mediated mechanism [48] or other immunomodulatory effects of statins. For the effect on vessel density within the tumor, we predict that statins ability to inhibit vessel formation in early disease was blunted by multiple mechanisms activated in advanced disease.

In conclusion, statins treatment leads to increase in Tregs infiltration within CRC tumor tissues which might be associated with CRC positive prognosis. In addition, statins decrease CRC angiogenesis. These findings suggest that statins reduce the risk of CRC.

## MATERIALS AND METHODS

### Study population

Institutional review board (IRB) approval was acquired beore conducting this research. This study was conducted on archived tumor samples acquired from the pathology department at King Abdullah Univeristy Hospital (KAUH). KAUH is a tertiary care center receiving cases with health problems, such as cancer, from other hospitals in Jordan. By reviewing records of paraffin-embeded CRC samples available between January 2008 till December 2015, a total of 200 samples were found. Unfortunately, medication records of patients referred from other hospitals were not available in KAUH archives. To confirm the status of statins use, investigators had to contact the 200 patients, one by one, to figure out if they are receiving statins, which statin and dose. Some other information were hard to be retrieved, such as duration of statin therapy. Some patients were dead and some changed their contact information, and eventually we ended up with 47 samples. Among those samples, 25 were from CRC patients who were using statins and 22 samples were from CRC patients who were not using statins as controls. Samples would be compared in respect to three features: Tregs infiltration, angiogenesis, and TGF-β_1_ content. Tumor samples were staged upon resection according to the American Joint Committee on Cancer’s TNM staging system [27] adopted around that time.

### Regulatory T Lymphocytes infiltration and angiogenesis

Immunohistochemistry was conducted to study both Tregs infiltration and angiogenesis.

### Immunohistochemistry

The specimens were analyzed immunohistochemically using sections from paraffin-embedded CRC tissue. The samples were analyzed for their infiltration of FoxP3-positive T cells, which is a marker for Tregs, and CD31. After a microtome sectioning from paraffin-embedded blocks using Leica rotary microtome, these sections (4 µm) were labeled, heated using oven at 75°C, deparafinized by dewaxing with xylene (two containers for 15 minutes in each) and hydrated with ethanol (100%, 100%, 90%, 80%, and 70%, for 2 minutes for each). After that, an antigen retrieval step was performed. Heat-induced antigen retrieval was done using PT-linked instrument (DAKO, Denmark) and high pH citrate buffer (DAKO, Denmark) for FoxP3 and low pH citrate buffer for CD31. Then, these sections were treated with 3% hydrogen peroxide (DAKO, Denmark) for 10 minutes in order to block endogenous peroxidase activity. Sections were then washed with Phosphate-buffered saline (PBS), and then incubated with primary antibodies, monoclonal anti-mouse FoxP3 (Abcam; 236A/E7, ab20034; Cambridge, MA; 1:200 dilution) and monoclonal anti-mouse CD31 antibody (Santa Cruz Biotechnology; sc-376764; 1:200 dilution), for 60 minutes at 4°C. This was followed by washing with PBS buffer and incubation with the secondary antibody, which is horseradish peroxidase (Envision Flex / HRP – DAKO, Denmark) for 20 minutes at 20°C. After washing the sections with PBS buffer, antigen signal detection was conducted by visualizing a color after a reaction with 3,3’ diaminobenzidine (DAB + chromogen; Dako Cytomation). Then, the slides were counter-stained with Mayer hematoxylin, dehydrated with ethanol (70%, 80%, 90%, and 100%, 2 minutes for each), dried in the oven for 3–5 minutes, immersed in xylene, and then mounted by DPX mounting media to be ready for visualizing under microscope. Positive control was tonsils tissue for FoxP3 and appendix tissue for CD31, while PBS buffer replaced the primary antibody was used as negative control.

Images were obtained at 400x magnification. The number of FoxP3+ cells and CD31+ blood vessels were counted by two investigators in 5 high power fields of each tumor section and then the mean number of these cells and blood vessels were calculated. The counting was performed by blinded investigators who did not participate in the immunohistochemical staining procedure and who had no knowledge of the clinical data about these samples.

### Transforming growth Factor-β content

TGF-β content was examined through three steps, proteins extraction, protein assay, and ELISA.

### Protein extraction

Protein extraction was performed using a commercial protein extraction kit (Qproteome FFPE Tissue Kit, Qiagen, Hilden, Germany; catalog number: 37623) according to manufacturer’s protocol. The procedure started with two sections of each sample that were cut from the tissue blocks (4 µm thick). Deparaffinization of the tissues was performed starting with incubating samples in an oven for 30 minutes at 70°C, then they were transferred to two containers of fresh xylene for 10 minutes in each. After that, samples were incubated in a series of decreasing ethanol concentrations (100%, 100%, 96%, 96%, 70% and 70%), for 10 minutes in each concentration and finally they were immersed in double-distilled water for 30 seconds. After tapping the slide on a paper towel, tissue samples were transferred into eppendorf tubes and mixed with 100 μL of extraction buffer EXB that is provided in the kit. Samples were incubated on ice for 5 minutes, and then incubated at 100°C in a heating block, followed by incubation at 80°C with shaking for 2 hours using a water bath shaker. At the end of incubation period, samples were centrifuged for 15 minutes at 14,000 g at 4°C to enable transferring the supernatant-containing proteins into new eppendorf tubes [28]. The tubes were then stored at –20°C until the time of analysis.

### Protein assay

Protein assay was performed to quantify the extracted protein. It was performed by using Microfuge Tube Assay Protocol of Reducing Agent-compatible/Detergent Compatible (RC DC) Protein assay (BioRad, catalog number: 500–0120). First step was A′ solution preparation by adding 5 μL of DC Reagent S to each 250 μL of DC Reagent A. Standard curve samples were prepared by preparing 5 serial dilutions of bovine serum albumin (BSA) as protein standards: 1.5 mg/mL, 0.75 mg/mL, 0.375 mg/mL, 0.1875 mg/mL, and 0.09385 mg/mL. After pipetting 25 μL of each standard and sample into clean dry microfuge tube, 125 of RC Reagent I were pipetted into each tube, and then these tubes were vortexed and incubated for 1 minute at room temperature. RC Reagent II was added by adding 25 μL to each tube, then the tubes were vortexed, and later centrifuged at 15,000 × g for 5 minutes. After that supernatants were discarded and 127 μL of A′ solution were added to each tube, vortexed, and incubated at room temperature until the precipitates were dissolved completely. This was followed by another vortex step, then 1 mL of DC Reagent B was added to each tube, vortexed, and incubated for 15 minutes at room temperature. The last step is absorbance reading at 750 nm. Absorbance for each sample was read in duplicate.

### ELISA

TGF-β_1_ concentration was determined by using a Human TGF-β_1_ ELISA Kit (MBS175889). This kit is based on a quantitative standard sandwich enzyme linked immune sorbent technique. The experiment was carried out according to manufacturer protocol. Solution A (1N HCl) was prepared by adding 8.35 mL of 12N HCl into 91.67 mL of deionized water. Solution B (1.2N NaOH/0.5M 4-(2-hydroxyethyl)-1-piperazineethanesulfonic acid (HEPES)) was prepared by adding 12 mL of 10N NaOH and 11.9 g of HEPES into 75 mL deionized water, then adding deionized water to adjust volume to 100 mL. Standards were prepared by adding 1 mL of standard diluent buffer into the tube that containing 10,000 pg/mL human TGF-β_1_ standard. 1000 pg/mL of human TGF-β1 standard was prepared by adding 0.1 mL of the first standard into 0.9 mL of sample diluent buffer, while the remaining standards (500 pg/mL, 250 pg/mL, 125 pg/mL, 62.5 pg/mL, 31.2 pg/mL, 15.6 pg/mL, and 7.8 pg/mL) were prepared by serial dilution.

At first, samples were diluted using deionized water to get 0.1 mg/mL dilution. Then these samples were activated (because TGF-β1 mostly contained in as inactive form in samples) by adding 20 μL of solution A to each 40 μL of each sample, 10 minutes later 20 μL of solution B were added to each sample. The 96-well plate was pre-coated with a mouse monoclonal antibody specific for TGF-β1. A total of 0.1 mL of blank, which is a sample diluent buffer, and 0.1 mL of each standard and sample were pipetted into the plate wells and incubated at 37°C for 90 minutes, each sample and standard was loaded in duplicate. After discarding the solution, 0.1 mL of biotinylated anti-human TGF-β1 antibody working solution, which is a goat polyclonal antibody that is specific for TGF-β1 and it was diluted with antibody diluent buffer to get a 1:100 dilution, were added into each well. The plate was incubated at 37°C for 60 minutes, then it was washed three times using PBS buffer. After discarding washing buffer 0.1 of ABC working solution, which is Avidin-Biotin-Peroxidase Complex that was diluted with ABC diluent buffer to get 1:100 dilution, were added into each well, and the plate was incubated at 37°C for 30 minutes. This was followed by five times washing using PBS buffer to wash away any unbound conjugates. 0.09 mL of TMP color developing agent, HRP substrate, were added to each well, and the plate was incubated at 37°C in dark for 25 minutes. Then 0.1 mL of acidic 3,3,5,5- tetramethylbenzidine (TMB) stop solution was added to each well. Finally, the absorbance was read at 450 nm using a microplate reader.

### Statistical analysis

Data analysis was performed using Graphpad Prism software version 7.0 (GraphPad Software, La Jolla, California, USA). D’Agostino & Pearson omnibus normality test indicated that data were not normaly distributed. For that reason, Mann-Whitney *U* nonparametric test was used to analyze the data. To study the effect of disease stage on FoxP3+ cells infiltration and tumor angiogenesis, we used first the two-way analysis of variance (ANOVA) test. For multiple comparison between all groups, we used Tukey’s multiple comparisons test. Dichotomization of some categorical variables was considered for statistical analysis of some study variables. This dichotomization was based on sample size and was performed in advance of conducting statistical analysis in order to avoid small sample size upon further stratification of data. Therefore, the TNM stage of CRC was dichotomized as early disease (stages I and II) and advanced disease (stages III and IV). Mann-Whitney *U* test was used to compare differences between groups within each disease stage. A difference between study groups was considered to be significant if *P*-value was < 0.05.

## Abbreviations

(CRC): Colorectal cancer
(Tregs): Regulatory T Lymphocytes
(CDK): cyclin-dependent kinase
(KAUH): King Abdullah Univeristy Hospital
(PBS): Phosphate-buffered saline
(DAB): 3,3′ diaminobenzidine
(RC DC): Reducing Agent-compatible/Detergent Compatible
(BSA): bovine serum albumin
(HEPES): 4-(2-hydroxyethyl)-1-piperazineethanesulfonic acid
(TMB): 3,3,5,5- tetramethylbenzidine
(ANOVA): analysis of variance

## References

[1] Siegel RL, Miller KD, Jemal A (2017). Cancer Statistics, 2017. CA Cancer J Clin.

[2] Al-Sayaideh A, Nimri O, Arqoub K, Al-Zaghal M, Halasa W (2012). Cancer incidence in Jordan-2012. Jordan Cancer Registry.

[3] Haggar FA, Boushey RP (2009). Colorectal cancer epidemiology: incidence, mortality, survival, and risk factors. Clin Colon Rectal Surg.

[4] Carethers JM, Stoffel EM (2015). Lynch syndrome and Lynch syndrome mimics: The growing complex landscape of hereditary colon cancer. World J Gastroenterol.

[5] Finn OJ (2008). Cancer immunology. N Engl J Med.

[6] Dunn GP, Old LJ, Schreiber RD (2004). The three Es of cancer immunoediting. Annu Rev Immunol.

[7] Koebel CM, Vermi W, Swann JB, Zerafa N, Rodig SJ, Old LJ, Smyth MJ, Schreiber RD (2007). Adaptive immunity maintains occult cancer in an equilibrium state. Nature.

[8] Becker JC, Andersen MH, Schrama D, Thor Straten P (2013). Immune-suppressive properties of the tumor microenvironment. Cancer Immunol Immunother.

[9] Jacobs JF, Nierkens S, Figdor CG, de Vries IJ, Adema GJ (2012). Regulatory T cells in melanoma: the final hurdle towards effective immunotherapy?. Lancet Oncol.

[10] Zou W (2006). Regulatory T cells, tumour immunity and immunotherapy. Nat Rev Immunol.

[11] Liu VC, Wong LY, Jang T, Shah AH, Park I, Yang X, Zhang Q, Lonning S, Teicher BA, Lee C (2007). Tumor evasion of the immune system by converting CD4+CD25- T cells into CD4+CD25+ T regulatory cells: role of tumor-derived TGF-beta. J Immunol.

[12] Xu P, Fan W, Zhang Z, Wang J, Wang P, Li Y, Yu M (2017). The Clinicopathological and Prognostic Implications of FoxP3(+) Regulatory T Cells in Patients with Colorectal Cancer: A Meta-Analysis. Front Physiol.

[13] Boudreau DM, Yu O, Johnson J (2010). Statin use and cancer risk: a comprehensive review. Expert Opin Drug Saf.

[14] Mansourian M, Haghjooy-Javanmard S, Eshraghi A, Vaseghi G, Hayatshahi A, Thomas J (2016). Statins Use and Risk of Breast Cancer Recurrence and Death: A Systematic Review and Meta-Analysis of Observational Studies. J Pharm Pharm Sci.

[15] Manthravadi S, Shrestha A, Madhusudhana S (2016). Impact of statin use on cancer recurrence and mortality in breast cancer: A systematic review and meta-analysis. Int J Cancer.

[16] Dawe DE, Mahmud S (2017). Biologic and epidemiologic evidence assessing if statins prevent prostate cancer. Can J Urol.

[17] Stopsack KH, Greenberg AJ, Mucci LA (2017). Common medications and prostate cancer mortality: a review. World J Urol.

[18] Haukka J, Niskanen L, Auvinen A (2017). Risk of Cause-Specific Death in Individuals with Cancer-Modifying Role Diabetes, Statins and Metformin. Int J Cancer.

[19] Ananthakrishnan AN, Cagan A, Cai T, Gainer VS, Shaw SY, Churchill S, Karlson EW, Murphy SN, Liao KP, Kohane I (2016). Statin Use Is Associated With Reduced Risk of Colorectal Cancer in Patients With Inflammatory Bowel Diseases. Clin Gastroenterol Hepatol.

[20] National Library of Medicine

[21] Chan KK, Oza AM, Siu LL (2003). The statins as anticancer agents. Clin Cancer Res.

[22] Rao S, Porter DC, Chen X, Herliczek T, Lowe M, Keyomarsi K (1999). Lovastatin-mediated G1 arrest is through inhibition of the proteasome, independent of hydroxymethyl glutaryl-CoA reductase. Proc Natl Acad Sci U S A.

[23] Dulak J, Jozkowicz A (2005). Anti-angiogenic and anti-inflammatory effects of statins: relevance to anti-cancer therapy. Curr Cancer Drug Targets.

[24] Weis M, Heeschen C, Glassford AJ, Cooke JP (2002). Statins have biphasic effects on angiogenesis. Circulation.

[25] Kochuparambil ST, Al-Husein B, Goc A, Soliman S, Somanath PR (2011). Anticancer efficacy of simvastatin on prostate cancer cells and tumor xenografts is associated with inhibition of Akt and reduced prostate-specific antigen expression. J Pharmacol Exp Ther.

[26] Goc A, Kochuparambil ST, Al-Husein B, Al-Azayzih A, Mohammad S, Somanath PR (2012). Simultaneous modulation of the intrinsic and extrinsic pathways by simvastatin in mediating prostate cancer cell apoptosis. BMC Cancer.

[27] Amin MB, Edge SB, American Joint Committee on Cancer (2017). AJCC cancer staging manual.

[28] Kroll J, Becker KF, Kuphal S, Hein R, Hofstadter F, Bosserhoff AK (2008). Isolation of high quality protein samples from punches of formalin fixed and paraffin embedded tissue blocks. Histol Histopathol.

[29] Rodriguez-Perea AL, Montoya CJ, Olek S, Chougnet CA, Velilla PA (2015). Statins increase the frequency of circulating CD4+ FOXP3+ regulatory T cells in healthy individuals. J Immunol Res.

[30] Mausner-Fainberg K, Luboshits G, Mor A, Maysel-Auslender S, Rubinstein A, Keren G, George J (2008). The effect of HMG-CoA reductase inhibitors on naturally occurring CD4+CD25+ T cells. Atherosclerosis.

[31] Tang TT, Song Y, Ding YJ, Liao YH, Yu X, Du R, Xiao H, Yuan J, Zhou ZH, Liao MY, Yao R, Jevallee H, Shi GP (2011). Atorvastatin upregulates regulatory T cells and reduces clinical disease activity in patients with rheumatoid arthritis. J Lipid Res.

[32] Meng X, Zhang K, Li J, Dong M, Yang J, An G, Qin W, Gao F, Zhang C, Zhang Y (2012). Statins induce the accumulation of regulatory T cells in atherosclerotic plaque. Mol Med.

[33] Vlad C, Kubelac P, Fetica B, Vlad D, Irimie A, Achimas-Cadariu P (2015). The prognostic value of FOXP3+ T regulatory cells in colorectal cancer. J BUON.

[34] Wang Q, Feng M, Yu T, Liu X, Zhang P (2014). Intratumoral regulatory T cells are associated with suppression of colorectal carcinoma metastasis after resection through overcoming IL-17 producing T cells. Cell Immunol.

[35] Ma YX, Li WH, Xie Q (2013). Rosuvastatin inhibits TGF-beta1 expression and alleviates myocardial fibrosis in diabetic rats. Pharmazie.

[36] Kim SI, Han DC, Lee HB (2000). Lovastatin inhibits transforming growth factor-beta1 expression in diabetic rat glomeruli and cultured rat mesangial cells. J Am Soc Nephrol.

[37] Li YB, Yin JJ, Wang HJ, Wang J, Tian H, Yang M (2011). Effect of simvastatin on expression of transforming growth factor-beta and collagen type IV in rat mesangial cells. Pharmacology.

[38] Chen ZX, Lei MX, Zhu LF, Zhang J (2005). [Effects of simvastatin on TGF-beta system of diabetic rat kidneys]. [Article in Chinese]. Zhong Nan Da Xue Xue Bao Yi Xue Ban.

[39] Porreca E, Di Febbo C, Baccante G, Di Nisio M, Cuccurullo F (2002). Increased transforming growth factor-beta(1) circulating levels and production in human monocytes after 3-hydroxy-3-methyl-glutaryl-coenzyme a reductase inhibition with pravastatin. J Am Coll Cardiol.

[40] Zhang D, Wang S, Guan Y, Wang L, Xie W, Li N, Zhao P, Su G (2011). Effect of oral atorvastatin on CD4+ CD25+ regulatory T cells, FoxP3 expression, and prognosis in patients with ST-segment elevated myocardial infarction before primary percutaneous coronary intervention. J Cardiovasc Pharmacol.

[41] Urbich C, Dernbach E, Zeiher AM, Dimmeler S (2002). Double-edged role of statins in angiogenesis signaling. Circ Res.

[42] Skaletz-Rorowski A, Walsh K (2003). Statin therapy and angiogenesis. Curr Opin Lipidol.

[43] Wang D, Li T, Wei H, Wang Y, Yang G, Tian Y, Zhao Z, Wang L, Yu S, Zhang Y, Chen J, Jiang R, Zhang JN (2016). Atorvastatin enhances angiogenesis to reduce subdural hematoma in a rat model. J Neurol Sci.

[44] Chaudagar KK, Mehta AA (2014). Effect of atorvastatin on the angiogenic responsiveness of coronary endothelial cells in normal and streptozotocin (STZ) induced diabetic rats. Can J Physiol Pharmacol.

[45] Siddiqui AJ, Gustafsson T, Fischer H, Widegren U, Hao X, Mansson-Broberg A, Grinnemo KH, Dellgren G, Sylven C (2004). Simvastatin enhances myocardial angiogenesis induced by vascular endothelial growth factor gene transfer. J Mol Cell Cardiol.

[46] Salama P, Stewart C, Forrest C, Platell C, Iacopetta B (2012). FOXP3+ cell density in lymphoid follicles from histologically normal mucosa is a strong prognostic factor in early stage colon cancer. Cancer Immunol Immunother.

[47] Bendardaf R, Buhmeida A, Hilska M, Laato M, Syrjanen S, Syrjanen K, Collan Y, Pyrhonen S (2008). VEGF-1 expression in colorectal cancer is associated with disease localization, stage, and long-term disease-specific survival. Anticancer Res.

[48] Kong F, Ye B, Lin L, Cai X, Huang W, Huang Z (2016). Atorvastatin suppresses NLRP3 inflammasome activation via TLR4/MyD88/NF-kappaB signaling in PMA-stimulated THP-1 monocytes. Biomed Pharmacother.

